# Development of a Multimodal, Physiotherapist-Led, Vocational Intervention for People with Inflammatory Arthritis and Reduced Work Ability: A Mixed-Methods Design Study

**DOI:** 10.1007/s10926-023-10170-y

**Published:** 2024-02-05

**Authors:** N. F. Bakker, S. F. E. van Weely, N. Hutting, Y. F. Heerkens, J. A. Engels, J. B. Staal, M. van der Leeden, A. Boonen, T. P. M. Vliet Vlieland, J. Knoop

**Affiliations:** 1 Department of Orthopaedics, Rehabilitation and Physical Therapy, Leiden University Medical Center, Albinusdreef 2, P.O. Box 9600, 2300 RC Leiden, The Netherlands; 2 Research Group Occupation & Health, HAN University of Applied Sciences, Nijmegen, The Netherlands; 3 Musculoskeletal Rehabilitation Research Group, HAN University of Applied Sciences, Nijmegen, The Netherlands; 4 Radboud Institute for Health Sciences, IQ Healthcare, Radboud University Medical Center, Nijmegen, The Netherlands; 5 Reade, Rehabilitation and Rheumatology, Amsterdam, The Netherlands; 6 Amsterdam Movement Sciences, Musculoskeletal Health, Amsterdam, The Netherlands; 7 Division of Rheumatology, Department of Internal Medicine, Maastricht University Medical Center, Maastricht, The Netherlands; 8 Care and Public Health Research Institute (CAPHRI), Maastricht University, Maastricht, The Netherlands

**Keywords:** Vocational rehabilitation, Physiotherapy, Rheumatoid arthritis, Axial spondyloarthritis, Work ability

## Abstract

**Purpose:**

Work ability of people with rheumatoid arthritis (RA) and axial spondyloarthritis (axSpA) is reduced, but underexamined as a clinical treatment target. The evidence on vocational interventions indicates that delivery by a single healthcare professional (HCP) may be beneficial. Physiotherapist (PT)-led interventions have potential because PTs are most commonly consulted by RA/axSpA patients in the Netherlands. The aim was to develop a PT-led, vocational intervention for people with RA/axSpA and reduced work ability.

**Methods:**

Mixed-methods design based on the Medical Research Council (MRC) framework for developing and evaluating complex interventions, combining a rapid literature review and six group meetings with: patient representatives (*n* = 6 and 10), PTs (*n* = 12), (occupational) HCPs (*n* = 9), researchers (*n* = 6) and a feasibility test in patients (*n* = 4) and PTs (*n* = 4).

**Results:**

An intervention was developed and evaluated. Patient representatives emphasized the importance of PTs’ expertise in rheumatic diseases and work ability. The potential for PTs to support patients was confirmed by PTs and HCPs. The feasibility test confirmed adequate feasibility and underlined necessity of training PTs in delivery. The final intervention comprised work-focussed modalities integrated into conventional PT treatment (10–21 sessions over 12 months), including a personalized work-roadmap to guide patients to other professionals, exercise therapy, patient education and optional modalities.

**Conclusion:**

A mixed-methods design with stakeholder involvement produced a PT-led, vocational intervention for people with RA/axSpA and reduced work ability, tested for feasibility and ready for effectiveness evaluation.

**Supplementary Information:**

The online version contains supplementary material available at 10.1007/s10926-023-10170-y.

## Introduction

Rheumatoid arthritis (RA) and axial spondyloarthritis (axSpA), two of the most common types of inflammatory arthritis (IA), are associated with joint pain, stiffness, fatigue [1, 2] and diminished health-related quality of life [1, 3]. Despite advancements in pharmacological treatments, people with RA or axSpA experience reduced work ability compared to the general population [4, 5]. They encounter both physical limitations and psychosocial obstacles in the workplace and they struggle to maintain a sustainable balance between work and activities of daily life [6–8]. This can lead to sick leave [9], decreased productivity during work (e.g. “presenteeism”) [10] or even job loss [4]. On average, 38% of people diagnosed with IA lose their jobs within the first years after diagnosis [11]. Reduced work ability incurs substantial costs for both individuals and society [3, 12]. In Europe, work-related costs connected to RA [13, 14] and axSpA [13, 14] were in 2015 estimated at €4000–€5000 per person per year.

Only a few studies addressed vocational (work-oriented) interventions for people with IA. Two recent systematic reviews [11, 15] identified six randomized controlled trials (RCTs) [16–21] and one pilot RCT [22] assessing the effects of supervised vocational interventions on work-related outcomes in IA. In two RCTs [17, 19], a multidisciplinary team delivered the intervention, while in the remaining five (pilot) RCTs a single healthcare professional (HCP) (i.e. occupational therapist (OT) [18, 21, 22], OT or physiotherapist (PT) [20] or rehabilitation counsellor [16]) delivered the intervention. In the two RCTs delivered by a multidisciplinary team [17, 19], no effects on work-related outcomes were found. In the five single HCP-led interventions, all consisting of multiple treatment modalities, favourable effects on various work-related outcomes, including work instability, work limitations and employment status, were reported [16, 18, 20–22].

Given the variability in HCPs delivering vocational interventions, the most appropriate profession for administering vocational interventions in RA/axSpA is unclear. Recently, a new study has been published describing the development of a multimodal, OT-led, vocational intervention for people with IA [23]. However, PT-led vocational interventions also could be promising in RA or axSpA, for several reasons. First, physical fitness and muscle strength are associated with employability [24, 25] and PTs are experts in providing exercise therapy aimed at gradually improving these factors. Second, PT-led vocational interventions have proven effective in enhancing work-related outcomes among other populations, such as individuals with hip and knee osteoarthritis [26, 27] and musculoskeletal pain [28, 29]. Third, in the Netherlands, PTs are relatively frequently consulted as HCPs, as they are found to be utilized by 25–50% of the people with IA on a yearly basis [30]. However, conventional PT treatments for people with RA or axSpA currently lack vocational interventions. For developing a PT-led vocational intervention, it is crucial to encompass insights of all relevant stakeholders in order to address the complexity of reduced work ability in people with RA or axSpA [31].

Therefore, the aim of this study was to develop a multimodal, PT-led vocational intervention aimed at improving work ability in people with RA or axSpA and reduced work ability. This study will address the following research question: Which treatment modalities, based on the literature and insights from stakeholders, should be included in a comprehensive vocational intervention led by PTs to optimize work ability of individuals with RA or axSpA who have reduced work ability.

## Methods

### Study Design

**Fig. 1:**
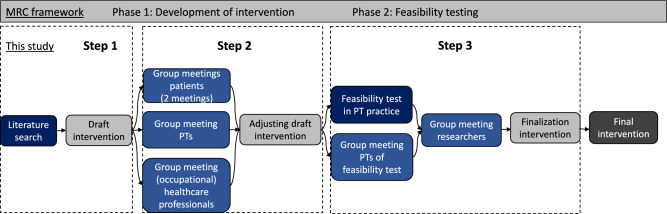
Flowchart of the intervention development process

Based on the Medical Research Council (MRC) framework [31], a non-linear approach for developing and evaluating complex interventions, a multimodal, PT-led, vocational intervention was developed. The MRC framework encourages a participatory design, with involvement of relevant stakeholders, including patient representatives, PTs, (occupational) HCPs and researchers, throughout all stages of intervention development. The MRC framework divides the development and evaluation process into four phases: (1) development of an intervention, (2) feasibility testing, (3) evaluation and (4) implementation. The present study only covers phases one and two; phases three and four will be described in other papers. Phase one comprised two steps: (1) a rapid literature review; (2) combining group meetings (qualitative data) with questionnaires (quantitative data) in multiple stakeholder groups. Phase two involved a feasibility test and a final group meeting evaluating the draft intervention (see Fig. 1). The reporting of qualitative aspects followed the COnsolidated criteria for REporting Qualitative research (COREQ) checklist [32]. The development process received ethical approval from the Medical Ethical Committee Leiden-Den Haag-Delft (METC-LDD; NL75919.058.20). All participants provided written informed consent.

### Study Setting and Context

In the Netherlands, employers are legally mandated to establish a contract with an occupational healthcare service [33]. Employees experiencing prolonged sick leave (>6 weeks) receive care from an occupational physician affiliated with such a service [34, 35]. Employees can also consult an occupational physician for work-related issues as a preventive measure. Self-employed people are expected to protect themselves against disability risk by purchasing disability insurance but, in practice, only around a quarter of these people do so [36]. Those lacking this insurance are not entitled to counselling or benefits in cases of sick leave or job loss.

The intervention in this study will be provided by primary care PTs. In the Netherlands, individuals have direct access to PT services. Since 2012, physiotherapy costs are not covered by basic health insurance but full or partial reimbursement can be obtained through supplementary health insurance; otherwise, patients must cover the expenses themselves. Approximately 76% of people with IA in the Netherlands have supplementary health insurance [30]. On average, people with IA utilize 25 physiotherapy sessions of 30 min per year [30].

#### Phase 1: Step 1 Literature Search

The objective of step 1 was to develop a draft intervention (version 1.0) based on published literature and the collective clinical experience of the research team. A rapid literature review was conducted encompassing the Dutch physiotherapy guidelines and recommendations [37, 38] and work ability management guidelines and recommendations for people with IA [39, 40]. The search was extended to PubMed to identify RCTs evaluating HCP-led vocational interventions for people with IA. Studies published in English or Dutch up until February 2021 were considered. The search terms included ‘inflammatory arthritis’, ‘RA’, ‘axSpA’ in conjunction with ‘vocational rehabilitation’, ‘work-oriented interventions’ and ‘occupational health’.

#### Phase 1: Step 2 Group Meetings with Stakeholders

Relevant stakeholders were engaged in group meetings to discuss the draft intervention (version 1.0). To ensure comprehensive representation, we employed purposive sampling, adhering to predefined inclusion criteria for each stakeholder group. We aimed for a balanced and diverse sample with regard to gender, age and, for patient representatives, diagnoses of RA or axSpA.

The group meetings had three objectives: to determine stakeholder requirements and preferences concerning the content of the PT-led, vocational intervention; to review and refine the content of the initial draft intervention (version 1.0); and to debate on the topics to be covered in the e-learning courses for participating PTs. Two weeks prior to each group meeting, participants received written information about the draft intervention (version 1.0), along with a concise questionnaire. Participants were instructed to review the draft intervention and identify strengths, weaknesses and redundant or missing elements, in the proposed protocol by completing the questionnaire. Responses were requested a week before the meeting, enabling the research team (SvW, JK, NB) to compile and summarize the results in advance. For each group meeting, the research team developed a semi-structured interview guide (see Appendix 1 for topics discussed in each meeting) with open-ended questions, following the recommendations of Krueger [41]. The interview guide commenced with broad questions concerning stakeholder requirements and preferences for intervention content, concluding with a discussion of the draft intervention. Group meetings offer the advantage of fostering dynamic interactions among participants, encouraging exploration and clarification of individual and shared perspectives [41].

The following four group meetings were held:

1. Two with patient representatives.
2. One with PTs.
3. One multidisciplinary, including (occupational) HCPs (e.g. rheumatologists, nurse specialists, labour experts, occupational therapists and occupational physicians).

Recruitment for each group was as follows:

1. Patient representatives with RA or axSpA were recruited through the websites of the Dutch Arthritis Society (ReumaNederland) and the Dutch axial SpA foundation (Stichting axiale SpA) between March and April 2021. Inclusion criteria were self-reported RA or axSpA and current or past employment or self-employment.
2. In March 2021, PTs were recruited through the newsletter of the Royal Dutch Society for Physical Therapy (Koninklijk Nederlands Genootschap voor Fysiotherapie; KNGF), within a professional network of PTs specializing in rheumatic and musculoskeletal diseases (ReumaNetNL; www.reumanetnl.nl), and via the LinkedIn profiles of the researchers (SvW, JK, NB). Eligibility criteria for PTs included practising as a PT and having experience in treating people with RA or axSpA.
3. (Occupational) HCPs were identified by the research team and invited via email in April 2021. These were considered eligible if practising in the rheumatology field or as an occupational HCP and having experience with the target population.

All group meetings occurred between March and September 2021, with online being the chosen format due to COVID-19 restrictions [Microsoft Teams (Microsoft Corporation, Redmond, WA)]. Audio recordings of all meetings were made for reference purposes. Each meeting lasted 90 min, guided by three members of the research team (SvW, JK, NB). SvW, with expertise in moderating group discussions, moderated the meetings following a semi-structured interview guide, ensuring active participation of all attendees. JK and NB took real-time notes during meetings. After each session, proposed adjustments to the draft intervention were discussed by the research team (SvW, JK, NB).

During each meeting, consensus was achieved on points to consider, potential bottlenecks and proposed adjustments to the intervention. Subsequently, based on insights from the meetings, adjustments were made to the draft intervention, resulting in version 2.0. Furthermore, an e-learning course for PTs was developed, focussing on the Dutch occupational healthcare system and relevant work-related laws and regulations.

#### Phase 2 Feasibility Testing of Draft Intervention (Version 2.0)

Ten primary care PTs affiliated with the ReumaNetNL network, known for their expertise in treating rheumatic and musculoskeletal diseases, were recruited to participate in the feasibility test of the draft intervention. Invitations were sent through personal emails, accompanied by information about the study’s design and the draft treatment protocol. Additionally, PTs were provided with login credentials for three e-learning courses: two focussing on the integration of work into PT treatment, and one offering a preliminary e-learning course on the Dutch occupational healthcare system and work-related laws and regulations. The first two had been previously developed in another research project [42]. Completion of each course was assessed through a knowledge evaluation test. When PTs passed one, they gained access to the next course. All tests had to be passed before PTs were permitted to deliver the intervention. To ensure comprehensive understanding of the draft treatment protocol and the execution of the feasibility test, PTs attended an online meeting with the research team (SvW, JK, NB). To efficiently collect feedback on all intervention components, each PT was assigned to test two components of the draft intervention over a 2-month period, in June and July 2021. The components were tested with (self-)employed people diagnosed with RA or axSpA who were experiencing work-related problems due to their condition. PTs were supplied with an online questionnaire for providing feedback on various aspects of the treatment protocol. Additionally, the people with RA/axSpA were given the opportunity to offer feedback on the content and feasibility of the draft intervention through an online questionnaire facilitated by their PT.

### Group Meeting with Participating PTs in the Feasibility Test

PTs engaging in the feasibility test of version 2.0 were invited to a group meeting whose purpose was to assess their experiences with the e-learning courses and the feasibility test and to discuss potential adjustments to the draft intervention. During this meeting, feedback provided by the PTs through the questionnaire was the foundation for the discussion. The content and feasibility of intervention components were discussed and proposed adjustments were summarized and later deliberated on in a meeting involving researchers.

### Advisory Group Meeting with Researchers

The objective of this meeting was to achieve a consensus on the necessary adjustments to version 2.0. Researchers with expertise in rheumatology and/or work participation were identified by the research team and invited via email to participate. Two weeks before this meeting, participants received information regarding the draft treatment protocol and a questionnaire. They were instructed to review the draft treatment protocol and provide feedback on its strengths and weaknesses in the questionnaire. Responses were requested a week in advance, enabling the research team to utilize these insights during the meeting, where consensus was reached on adjustments to the protocol.

### Data Analysis of Group Meetings

To analyse qualitative data from group meetings, we used elements of thematic analysis [43]. During group meetings, two researchers (NB and JK) took notes, formulated points to consider, identified potential bottlenecks and proposed adjustments to the draft intervention. Subsequently, these notes were shared with participants, who were asked to check them for possible misinterpretations and suggest necessary adjustments (i.e. member checking [44]). This validation technique is generally used to enhance the accuracy, credibility, validity and transferability of qualitative data [45–47]. Following each group meeting, audio recordings were analysed by one researcher (NB). This process involved listening to recordings, cross-referencing them with the notes and verifying the completeness of the data analysis.

## Results

### Phase 1: Step 1

#### Literature Search

The literature search resulted in the identification of an international clinical guideline focussing on axSpA treatment [48], a Dutch clinical guideline for physiotherapy in RA [37], recommendations for physiotherapy in axSpA [38], a Dutch clinical guideline on addressing work participation in RA [39], Dutch recommendations on addressing work participation in IA [40], and seven RCTs investigating HCP-led vocational interventions for people with IA [16–22]. The Dutch physiotherapy guidelines and recommendations [37, 38] highlighted the role of PTs in counselling patients to prevent or reduce work-related problems, primarily through exercise therapy and providing information. The work participation guideline and recommendations [39, 40] stressed the need to involve all relevant stakeholders, including employers, HCPs and occupational HCPs, in preventing work disability. Seven RCTs evaluated the effects of supervised vocational interventions for IA [16–22]. Five reported positive effects for various work-related outcomes; these studies concerned interventions that were multimodal and included educational or vocational advice, such as information on rheumatic diseases and work-related laws and regulations [16, 18, 20–22]. Some also offered support for workplace accommodations, such as modifications to work environment or tasks. Three studies included optional workplace examinations [18, 21, 22], while none incorporated exercise therapy. Drawing upon these guidelines, recommendations and research findings, the initial draft intervention (version 1.0) was developed.

#### Draft Intervention (Version 1.0)

The initial draft was designed to incorporate work-focussed modalities into conventional physiotherapy treatment. It envisaged 10–21 PT sessions of 30 min, over a 12-month period. These could be delivered face-to-face, online or via telephone, or a combination of these. The number of sessions would depend on the level of health insurance coverage and participant needs and preferences. The intervention was structured into four steps:

1. *Assessment (first session)*: The participant and PT jointly identified and explored work-related limitations associated with RA/axSpA.
2. *Personalized treatment plan (second session)*: A tailored multimodal treatment plan was developed based on the participant’s needs.
3. *Execution of the treatment plan* (4–10 sessions within the first 3 months): Implementation of the treatment plan primarily face-to-face.
4. *Monitoring of the treatment plan* (4–9 ‘booster’ sessions within the following 9 months): Ongoing monitoring and support provided through face-to-face, online or telephone-based sessions.

The intervention incorporated three mandatory and two optional treatment modalities. *Mandatory modalities were*: (1) ‘Work-roadmap’; and (2) Exercise therapy, including a personalized physical activity plan; (3) Education and self-management support. *Optional modalities were*: (1) Online self-management course, administered by an experienced coach; and (2) Workplace examination conducted by the PT.

The content of these modalities was tailored to meet individual needs and preferences, with a focus on defined work-related treatment goals established through a semi-structured dialogue with the PT. See ‘Content of the final intervention’ for further details of this.

### Phase 1: Step 2 Group Meetings with Stakeholders

Thirty-seven participants took part in four group meetings (Table 1), with purposive sampling in accordance with the inclusion criteria. In the first two, 16 patient representatives participated (*n* = 4 (25%) with RA and *n* = 12 (75%) with axSpA), 14 of whom (88%) were currently employed. The third comprised 12 PTs, with an average professional experience of 23 years (SD 10). The fourth comprised nine (occupational) HCPs: six HCPs, including two rheumatologists, three specialized rheumatology nurses and one physician assistant and three occupational HCPs, including a labour expert, an OT and an occupational physician (in training).

**Table 1:** Characteristics of the participants in all steps of the study

Participant ID	No. of group meeting	Gender	Age (years)	Diagnosis	Disease duration (years)	(Self-)employed?	Work-related problems due to RA/axSpA?	Work-related problems due to RA/axSpA in the past?
*Patient representatives of group meeting 1 (n = 16)*								
1	1	Female	57	RA	30	Yes	No	Yes
2	1	Female	57	RA	7	No	n/a	Yes
3	1	Female	55	RA	26	Yes	No	Yes
4	1	Female	37	axSpA	9	Yes	No	Yes
5	1	Female	55	RA	20	Yes	Yes	Yes
6	1	Male	57	axSpA	14	Yes	Yes	Yes
7	2	Male	66	axSpA	42	Yes	No	No
8	2	Male	64	axSpA	4	Yes	Yes	Yes
9	2	Female	38	axSpA	4	Yes	No	Yes
10	2	Male	67	axSpA	42	No	n/a	Yes
11	2	Female	59	axSpA	37	Yes	Yes	Yes
12	2	Female	59	axSpA	36	Yes	Yes	Yes
13	2	Male	53	axSpA	31	Yes	No	Yes
14	2	Male	55	axSpA	30	Yes	No	Yes
15	2	Female	52	axSpA	21	Yes	Yes	Yes
16	2	Female	49	axSpA	14	Yes	No	Yes
*Patient representatives of the feasibility test (n = 4)*								
17	–	Male	58	axSpA	n/a	Yes	Yes	n/a
18	–	Male	40	axSpA	n/a	Yes	Yes	n/a
19	–	Female	51	RA	n/a	Yes	Yes	n/a
20	–	Male	32	axSpA	n/a	Yes	Yes	n/a

Participant ID	No. of group meeting	Gender	Age (years)	No. of years experience as PT	No. of new patients with RA/axSpA in treatment per year		Additional training followed on rheumatology or work ability?	
*Physiotherapists (n = 12)*								
21	3	Female	54	36	Missing		Yes, specialized occupational physiotherapist	
22	3	Male	51	30	7		Yes, additional rheumatology course	
23	3	Female	46	17	11		No	
24	3	Female	45	22	missing		No	
25	3	Female	52	24	8		No	
26	3	Female	55	30	7–30		Yes, additional work-related course	
27	3	Female	27	3	5		No	
28	3	Female	57	31	4		Yes, additional work-related course	
29	3	Female	59	36	10		No	
30	3	Female	35	13	7		Yes, additional rheumatology course	
31	3	Male	42	20	2		Yes, specialized occupational physiotherapist	
32	3	Female	42	11	Missing		Yes, specialized occupational physiotherapist	
*Physiotherapists of the feasibility test (n = 4)*								
33	6	Male	41	18	100		Yes, additional rheumatology courses	
34	6	Female	38	14	12		Yes, additional rheumatology and work-related courses	
35	6	Female	49	9	4		Yes, additional rheumatology course	
36	6	Female	57	35	6		Yes, additional rheumatology course	

Participant ID	No. of group meeting	Gender	Age (years)	Profession	Self-reported expertise rating (0–10) Rheumatology		Self-reported expertise rating (0–10) Work ability	
*Healthcare professionals (n = 6)*								
37	4	Female	58	Rheumatologist	8		8	
38	4	Female	53	Rheumatologist	9		2	
39	4	Female	61	Specialized rheumatology nurse	8		6	
40	4	Female	56	Specialized rheumatology nurse	8		3	
41	4	Female	54	Specialized rheumatology nurse	8		5	
42	4	Male	47	Physician assistant	8		6	
*Occupational healthcare professionals (n = 3)*								
43	4	Female	55	Labour expert	8		9	
44	4	Male	missing	Occupational physician (in training)	8		8	
45	4	Female	51	Occupational therapist	9		7	
*Researchers (n = 6)*								
46	5	Male	57	Professor	3		8	
47	5	Male	27	Junior researcher	5		8	
48	5	Male	53	Senior researcher	6		8	
49	5	Male	57	Senior researcher	5		9	
50	5	Female	32	Senior researcher	2		6	
51	5	Male	36	Senior researcher	3		8	

*n/a* not requested of participants in this meeting

During the two meetings involving patient representatives, several adjustments to the draft (version 1.0) were suggested (see Table 2). These pertained to the recruitment of PTs, emphasizing their knowledge and expertise in IA, as well as highlighting the importance of providing training to PTs on work ability and laws and regulations. Patient representatives emphasized the potential for PTs to support patients dealing with work-related problems. In the subsequent meeting with PTs, the potential importance of conducting a workplace examination and adopting a multidisciplinary approach was raised. PTs stressed the need for additional training on work-related laws and regulations and recognized the value of the ‘work-roadmap’. In the meeting with (occupational) HCPs, it was proposed to send personalized endorsement letters from the rheumatologists to participants in the intervention group, underscoring their support for the intervention. The importance of training PTs on work-related laws and regulations was reaffirmed. Therefore, drawing from relevant Dutch work-related laws and regulations [33, 34], as well as leveraging the expertise of the research team, a concept e-learning course was developed on the Dutch occupational healthcare system and work-related laws and regulations.

**Table 2:** Description of findings from all group meetings

Points to consider	Potential bottlenecks	Proposed adjustments to draft intervention	Adjusted elements in final intervention (version 3.0)
*Group meetings with patient representatives (n* = *16), concerning intervention 1.0*			
• PTs need additional training and expertise in treating people with IA	• Lack of knowledge on IA causes misunderstanding of people diagnosed with IA	• Select PTs on their expertise and knowledge on IA or otherwise educate PTs on IA	• The selection of PTs in the trial is primarily focused on knowledge and expertise in IA. If a PT has no knowledge on this topic, they will be educated in advance
• There is a high potential for a coordinator role by PTs to support participants in case of work-related limitations	• People with IA sometimes get lost in the web of (occupational) HCPs. PTs need a network of (occupational) HCPs nearby to guide the patient in how to receive care if necessary	• The ‘work-roadmap’ seems to be important and should be expanded	• The ‘work-roadmap’ was expanded and described in more detail in the protocol
• PTs generally lack expertise on options for support in case of work-related limitations	• PTs are not specifically trained to support people in case of work-related limitations	• Educate participating PTs on providing work-related care and laws and regulations and guide them in possible support options in case of work-related limitations. The ‘work-roadmap’ could be a tool to guide PTs	• An e-learning on work-related laws and regulations for PTs was developed
• Informing the employer and colleagues about the diagnosis can provide more understanding and options to adjust the job	• Some patients do not want to inform the employer and colleagues about the diagnosis because they are afraid to be differently approached or even dismissed	• Encourage, but do not require, informing the employer and colleagues about the diagnosis	• The topic of informing the employer about the diagnosis is added to the training of the PTs. Participants are not required to inform their employer
*Group meeting with physiotherapists (n* = *12), concerning intervention 1.0*			
• Workplace examination is important, consider making it a mandatory part of the intervention	• Some patients do not want to inform the employer and colleagues about the diagnosis, therefore it is not eligible to require a workplace examination	• Encourage, but do not require, a workplace examination	• The potential importance of a workplace examination was emphasized in the protocol
• PTs generally lack knowledge on work-related laws and regulations. It is important to educate participating PTs about this topic	• Lack of knowledge on work-related laws and regulations can cause wrong advice about job retention	• Educate participating PTs on work-related laws and regulations	• An e-learning on work-related laws and regulations for PTs was developed
• A multidisciplinary approach could be useful in case of work-related limitations. The ‘work-roadmap’ could therefore be a key modality of the intervention. However, the ‘work-roadmap’ needs to be expanded and described more detailed in the protocol	• PTs are not used to refer patients to occupational HCPs, therefore the ‘work-roadmap’ needs to be clear and precise	• The ‘work-roadmap’ needs to be expanded and described more detailed in the protocol and PTs need training in how to use the ‘work-roadmap’	• The ‘work-roadmap’ was expanded and described in more detail in the protocol
*Group meeting with (occupational) healthcare professionals (n* = *9), concerning intervention 1.0*			
• PTs generally lack knowledge on work-related laws and regulations. It is important to educate participating PTs about this topic	• Lack of knowledge on work-related laws and regulations can cause wrong advice about job retention	• Educate participating PTs on work-related laws and regulations	• An e-learning on work-related laws and regulations for PTs was developed
• It may be important for participants that their rheumatologist underline the importance of the intervention	• Participants may start with more confidence with the intervention if their rheumatologist underline the importance of the intervention	• The participant’s rheumatologist could be asked to sign a personalized endorsement letter to underline their support for the intervention. This letter should only be send to the participants that receive the intervention	• A personalized endorsement letter from the rheumatologist was added for participants in the intervention group. The rheumatologists signed the letter concurrently with providing the disease characteristics. Participants received the letter after randomization in the intervention group
*Group meeting with the physiotherapists of the feasibility test (n* = *3), concerning intervention 2.0*			
• PTs should be aware that going through the protocol and following the e-learning courses requires a large investment of time. It might be helpful to guide them through the protocol in a face-to-face session	• Insufficient information in advance about the time that must be invested in going through the protocol and following the e-learning courses can cause ill-prepared PTs	• A (online) face-to-face session about the study protocol and question hour will help PTs to understand the purpose of the protocol	• An online face-to-face session about the protocol and question hour was added to the mandatory training for PTs
• The readability of the protocol could be improved by adding a schematic overview of the intervention’s timeline instead of only describing this	• A schematic overview helps to look up things easily	• Add a schematic overview of the intervention’s timeline to the protocol	• A schematic overview of the intervention’s timeline was added to the protocol
*Group meeting with researchers (n* = *6), concerning intervention 2.0*			
• Workplace examination is important, emphasize this modality in the protocol	• The optionality of the workplace examination is appropriate because not all participants are open to discuss their diagnosis at work	• Emphasize the value of a workplace examination in the protocol and in the communication with PTs	• The value of a workplace examination was emphasized in the protocol and in communication between the PTs of the trial and the research team
• PTs need knowledge concerning work-related laws and regulations and the use of the protocol needs to be discussed to apply the intervention properly	• PTs need to be supported during the preparations (following e-learning courses and studying the protocol) by the research team to be good prepared for applying the intervention	• A (online) face-to-face session about the protocol and question hour will help PTs to understand the purpose of the protocol	• An online face-to-face session about the protocol and question hour was added to the mandatory training for PTs

#### Adjusting the Draft Intervention

In response to insights from stakeholder meetings, several adjustments were made to the draft intervention (Table 2). These included:

1. The ‘work-roadmap’ was expanded with more details to provide greater clarity and guidance.
2. The potential importance of a workplace examination, either by visiting the workplace or by examining pictures or videos, was emphasized in the protocol. This part was not mandatory due to patient representatives’ concerns, being that having to disclose their diagnosis to employers, supervisors or colleagues could be a barrier to participation. Some participants might fear that they would be treated differently or even dismissed.
3. Personalized endorsement letters from rheumatologists were integrated into the intervention. These letters were requested concurrently with the provision of disease characteristics (e.g. diagnosis, medication use). Participants received the letter after randomization into the intervention group.
4. An e-learning course was developed to educate PTs on the Dutch occupational healthcare system and work-related laws and regulations.

### Phase 2: Feasibility Testing of the Draft Intervention

Four PTs and their patients participated and provided feedback through questionnaires, subsequently discussed in a group meeting. See Table 1 for characteristics of these participants.

#### Group Meeting with PTs Participating in the Feasibility Test

Three of the four PTs involved in testing the draft intervention participated in the meeting. This, in combination with the feedback gathered from the PTs’ and patients’ questionnaires, led to the identification of several points to consider, potential bottlenecks, and proposed adjustments to the intervention (Table 2). Both PTs and patients found the protocol feasible. The PTs noted that executing the protocol and engaging with the e-learning courses demanded a significant time commitment. PTs should be made aware of this, and it might be beneficial to offer pre-trial guidance to them through a face-to-face session, possibly online. Additionally, the PTs suggested that the protocol’s readability could be enhanced by incorporating a schematic overview of the intervention’s timeline.

#### Group Meeting with Researchers

Six researchers with expertise in the fields of rheumatology and/or work ability participated. See Table 1 for their characteristics. They emphasized the significance of workplace examinations and recommended highlighting this in both the protocol and communication with PTs. The researchers stressed the importance of providing training to PTs as with the PTs participating in the feasibility test, they suggested including a face-to-face session on the treatment protocol, allowing participating PTs to ask questions directly to the research team (Table 2).

#### Finalization of the Intervention

Following the feasibility test and insights gained during the meeting with researchers, the intervention was finalized. The last round of adjustments incorporated the following key elements:

1. A schematic overview of the intervention’s timeline was integrated into the protocol.
2. The value of conducting a workplace examination was emphasized both within the protocol and in the communication between PTs participating in the trial and the research team.
3. An online face-to-face session intended to discussing the protocol and facilitating a question-and-answer period was included as a mandatory part of the training for participating PTs.

**Fig. 2:**
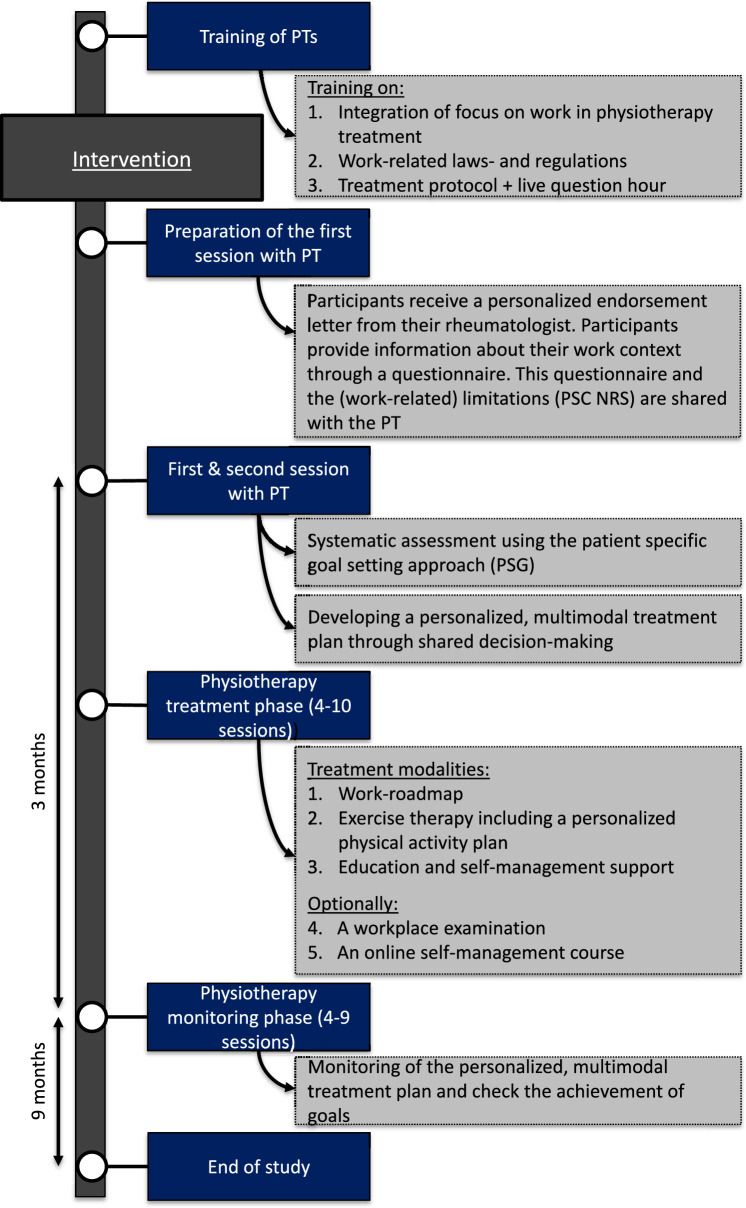
Overview of the finalized intervention

In Fig. 2, a schematic overview of the finalized intervention is displayed.

#### Content of the Finalized Intervention

The finalized intervention comprises 10–21 physiotherapy sessions of 30 min, delivered over a 12-month period. The 3-month treatment phase comprises 4–10 face-to-face sessions and the subsequent 9-month monitoring phase span 4–9 sessions, provided either face-to-face, online, via telephone, or a combination of these. The number of sessions depends on the participant’s needs and preferences but may also be related to the level of health insurance coverage for physiotherapy. The intervention comprises four steps:

*Step 1:* *Unravelling the participant’s work-related problems in relation to RA/axSpA.*

*Preparation of the first face-to-face consultation with the PT:*

The participant provides information about their work context using parts of a previously developed questionnaire [8]. Additionally, the participant lists three specific (work-related) limitations in physical activities, measured with the Patient Specific Complaints (PSC NRS) [49]. The questionnaire and (work-related) limitations in physical activities are shared with the PT prior to the first consultation.

*First consultation*: The PT conducts a systematic assessment, incorporating elements of motivational interviewing and using the patient-specific goal-setting approach (PSG) [50], to define in conjunction with the participant relevant work treatment goals aligned with the previously formulated specific (work-related) limitations in physical activities.

*Step 2: Developing a personalized, multimodal plan*

*Second consultation:*

In a shared decision-making dialogue, the PT and participant formulate a personalized treatment plan, including treatment modalities and supervision frequency, tailored to the participant’s needs and preferences. A decision tree is available to guide PTs in personalizing the intervention.

*Step 3: Conducting the personalized plan (* ***4–10 face-to-face sessions within 3 months*** *). The intervention comprises the following treatment modalities:*

1. *‘Work-roadmap’*:

A systematic scheme guides the PT in referring the participant to the appropriate (occupational) HCP if additional support is needed for work-related problems. The step-by-step roadmap is customized for each participant, based on the work context and the identified work-related limitations in physical activities. The PT serves as a coach, supporting the participant in taking actions according to the roadmap, with a coordinating role where multiple (occupational) HCPs are involved.

- *Optionally*, if considered beneficial by PT and participant: a workplace examination is conducted to address necessary adaptations at the workplace or to facilitate communication between the employee and supervisor or colleagues. This involves the PT visiting the workplace or examining pictures or videos of the participant working at the workplace.

2. *Exercise therapy including a personalized physical activity plan:*

A personalized physical activity plan is developed, based on physiotherapy guidelines for RA [37] and recommendations for axSpA [38], consisting of guided exercise therapy and exercises at home. These specifically target the work-related limitations identified by the PSC NRS and focus on improving modifiable factors for physical fitness, such as muscle strength, aerobic capacity, mobility, balance and neuromotor control, that have been linked to improved work ability [24, 25]. This plan is collaboratively developed by the PT and the participant and aims to integrate physical activity sustainably into the participant’s daily life. A behavioural graded activity approach is included if indicated and agreed upon [51].

3. *Education and self-management support:*

The PT provides personalized information on self-management strategies, addressing topics concerning work-related problems that people with RA/axSpA may experience. These include managing the balance between work and daily life demands and physical capacity, coping with fatigue, pain and energy level and discussing work disability with colleagues.

- *Optionally*, if considered beneficial by PT and participant: the participant can access an online self-management course to further enhance self-management and empowerment skills, specifically focussing on work-related issues. This course consists of two 1.5-h sessions, is paid from the study budget, and is organized and supervised by an experienced coach in rheumatic diseases and work-related problems. This course is organized in groups of four to six participants in the intervention group.

*Step 4: Monitoring phase (* ***4–9 ‘booster’ sessions within 9 months*** *)*

This phase is designed to facilitate and guide adherence to the treatment plan and assess the achievement of treatment goals.

## Discussion

Little research has been done on vocational interventions for people diagnosed with RA or axSpA. This study combined conventional physiotherapy modalities with specific work-oriented approaches into an intervention for people with RA or axSpA. During the development process, all potentially effective treatment modalities were integrated into a draft intervention and subsequently adapted in collaboration with relevant stakeholders. Furthermore, the intervention’s feasibility was tested by PTs and their patients, resulting in a feasible intervention and associated e-learning courses that are currently undergoing evaluation for both effectiveness and cost-effectiveness in an RCT.

The development process identified several points to consider to improve the intervention. Patient representatives identified a potential role for PTs in guiding patients to find appropriate (occupational) HCPs for their work-related problems. This potential coordinating role for PTs, aimed at assisting people with IA in navigating the (occupational) healthcare system, has also been identified in Danish research [7]. Patients emphasized the importance of the possession of specific expertise in rheumatic diseases and knowledge on the Dutch occupational healthcare system and work-related laws and regulations by PTs to their successful provision of the intervention. The need for extending this knowledge and expertise among participating PTs in the trial was the most frequently mentioned suggestion for improving the intervention. Additional e-learning courses have therefore been developed and tested for feasibility.

Depending on the national healthcare system and organization of occupational healthcare delivery, the most suitable HCP to deliver a vocational intervention may vary across countries. In the Netherlands, PTs as well as other professionals, including OTs, specialist rheumatology nurses, occupational health nurses or specialist occupational PTs, could also be considered for delivering such interventions. Accordingly, the parallel development and planned evaluation of an OT-led vocational intervention is very interesting [23]. In our study, generalist PTs, preferably with specific expertise in IA, were chosen to deliver the intervention for two reasons. First, exercise therapy may be an effective treatment modality to improve employability [24, 25]. Second, patient representatives repeatedly asserted expertise in IA to be even more important than expertise in work-related topics, since the latter can be more easily taught.

The number of physiotherapy sessions (10–21 during a 12-month follow-up period) may seem relatively large compared with other vocational intervention studies in IA (with numbers in the range 1–12) [16–22]. However, in the Netherlands, people with IA attend on average 25 physiotherapy sessions per year [30] and these do not specifically include the treatment of work-related problems. Madsen et al. will evaluate a vocational intervention comprising a similar number of sessions (9–18 in a 6-month period) [23]. Our rationale for such a large number of sessions is to enable the PT to (1) gradually increase the intensity of the exercise therapy, (2) monitor whether participants succeed in making sustainable lifestyle changes, (3) efficiently refer participants to other (occupational) HCPs and (4) monitor the impact of any work-related adaptations. Though more expensive than interventions with fewer sessions, we anticipate that the benefits in terms of savings for the healthcare system, due to reduced productivity loss, sick leave and disability benefits, will exceed these costs.

A strength of this study is the use of the systematic MRC framework to develop and test the intervention. With the exception of the planned study by Madsen et al. [23], none of the previous RCTs on vocational interventions in IA [16–22] reported on the development of interventions or used such a systematic framework. Other strengths of our study include the participation of patient representatives (*n* = 20) and a broad spectrum of relevant stakeholders, bringing different perspectives to the intervention’s content and proposed adjustments to the protocol. Other MRC framework-based studies in IA, though not focussed on vocational interventions, have included between 2 and 12 patient representatives [52, 53]. The participatory, mixed-methods design of this study contributes to the feasibility of the intervention, enhancing the chances of developing an intervention that meets the needs and requirements of end-users.

A limitation of this study is the relatively small sample size and short duration of the feasibility test. Nevertheless, we managed to identify important points for improving the intervention. Another limitation could be related to the analysis of group meeting data. We pragmatically used the member checking approach as opposed to transcribing and thematically analysing qualitative data. However, multiple studies have reported that alternative approaches in analysing qualitative interview data, like member checking, can generate valid results and maintain scientific rigour [46, 47]. It is likely that we reached data saturation because the same topics were identified and discussed in subsequent group meetings.

Another limitation is that the knowledge of participating PTs was assessed only after completion of each e-learning course. It might have been useful to have assessed their knowledge before the courses to see if it had improved. We chose not to do so to avoid raising an extra barrier to their participation. Finally, it is unclear whether the results of our trial can be generalized to other countries because this study was designed within the context of the Dutch healthcare and occupational healthcare system.

## Conclusion

Through an extensive collaborative process involving all relevant stakeholders and a feasibility test, we have developed a multimodal, PT-led, vocational intervention tailored for people with RA or axSpA and reduced work ability. This study contributes to the currently scarce knowledge on HCP-led vocational interventions by introducing a PT-led intervention combining physiotherapy modalities, e.g. exercise therapy and patient education, with specific work-oriented modalities (including a scheme for referring to (occupational) HCPs), and workplace examinations aimed at improving the work ability of people with RA or axSpA. The effectiveness and cost-effectiveness of this intervention will be evaluated through an RCT entitled ‘Physiotherapy WORKs!’.

## Supplementary Information

Below is the link to the electronic supplementary material.

Supplementary file1 (DOCX 18 kb)

## Data Availability

The data generated during this study will not be publicly available, but will be available upon reasonable request to the corresponding author.

## References

[1] Sieper J, Poddubnyy D. Axial spondyloarthritis. Lancet. 2017;390(10089):73–84. 28110981 10.1016/S0140-6736(16)31591-4

[2] Smolen JS, Aletaha D, McInnes IB. Rheumatoid arthritis. Lancet. 2016;388(10055):2023–2038. 27156434 10.1016/S0140-6736(16)30173-8

[3] Boonen A, Severens JL. The burden of illness of rheumatoid arthritis. Clin Rheumatol. 2011;30(Suppl 1):S3–S8. 21359507 10.1007/s10067-010-1634-9

[4] Berner C, et al. Work ability and employment in rheumatoid arthritis: a cross-sectional study on the role of muscle strength and lower extremity function. Int J Rheumatol. 2018;2018:3756207. 30154855 10.1155/2018/3756207 PMC6093007

[5] Nikiphorou E, Ramiro S. Work disability in axial spondyloarthritis. Curr Rheumatol Rep. 2020;22(9):55. 32719993 10.1007/s11926-020-00932-5 PMC7385005

[6] Hoving JL, et al. Work participation and arthritis: a systematic overview of challenges, adaptations and opportunities for interventions. Rheumatology (Oxford). 2013;52(7):1254–1264. 23472042 10.1093/rheumatology/ket111

[7] Madsen CMT, et al. Perceived challenges at work and need for professional support among people with inflammatory arthritis—a qualitative interview study. Scand J Occup Ther. 2023;30(5):640–649. 34644224 10.1080/11038128.2021.1989483

[8] de Buck PD, et al. A multidisciplinary job retention vocational rehabilitation programme for patients with chronic rheumatic diseases: patients’ and occupational physicians’ satisfaction. Ann Rheum Dis. 2004;63(5):562–568. 15082488 10.1136/ard.2003.007260 PMC1754975

[9] Lenssinck M-LB, et al. Consequences of inflammatory arthritis for workplace productivity loss and sick leave: a systematic review. Ann Rheum Dis. 2013;72(4):493–505. 23264343 10.1136/annrheumdis-2012-201998

[10] Verstappen SM. Rheumatoid arthritis and work: the impact of rheumatoid arthritis on absenteeism and presenteeism. Best Pract Res Clin Rheumatol. 2015;29(3):495–511. 26612244 10.1016/j.berh.2015.06.001

[11] Madsen CMT, et al. A systematic review of job loss prevention interventions for persons with inflammatory arthritis. J Occup Rehabil. 2021;31(4):866–885. 33782815 10.1007/s10926-021-09972-9

[12] Boonen A, van der Linden SM. The burden of ankylosing spondylitis. J Rheumatol Suppl. 2006;78:4–11. 17042055

[13] Raciborski F, Kłak A, Kwiatkowska B. Indirect costs of rheumatoid arthritis. Reumatologia. 2015;53(5):268–275. 27407258 10.5114/reum.2015.55830 PMC4847321

[14] Harvard S, et al. Costs of early spondyloarthritis: estimates from the first 3 years of the DESIR cohort. RMD Open. 2016;2(1):e000230. 27099778 10.1136/rmdopen-2015-000230 PMC4823587

[15] Butink MHP, et al. Non-pharmacological interventions to promote work participation in people with rheumatic and musculoskeletal diseases: a systematic review and meta-analysis from the EULAR taskforce on healthy and sustainable work participation. RMD Open. 2023;9(1):e002903. 36596655 10.1136/rmdopen-2022-002903 PMC10098260

[16] Allaire SH, Li W, LaValley MP. Reduction of job loss in persons with rheumatic diseases receiving vocational rehabilitation: a randomized controlled trial. Arthritis Rheum. 2003;48(11):3212–3218. 14613285 10.1002/art.11256

[17] de Buck PD, et al. Randomized comparison of a multidisciplinary job-retention vocational rehabilitation program with usual outpatient care in patients with chronic arthritis at risk for job loss. Arthritis Rheum. 2005;53(5):682–690. 16208658 10.1002/art.21452

[18] Macedo AM, et al. Functional and work outcomes improve in patients with rheumatoid arthritis who receive targeted, comprehensive occupational therapy. Arthritis Rheum. 2009;61(11):1522–1530. 19877106 10.1002/art.24563

[19] van Vilsteren M, et al. Effectiveness of an integrated care intervention on supervisor support and work functioning of workers with rheumatoid arthritis. Disabil Rehabil. 2017;39(4):354–362. 27097657 10.3109/09638288.2016.1145257

[20] Keysor JJ, et al. Efficacy of a work disability prevention program for people with rheumatic and musculoskeletal conditions: a single-blind parallel-arm randomized controlled trial. Arthritis Care Res (Hoboken). 2018;70(7):1022–1029. 28941189 10.1002/acr.23423 PMC6374024

[21] Baldwin D, et al. Randomized prospective study of a work place ergonomic intervention for individuals with rheumatoid arthritis and osteoarthritis. Arthritis Care Res (Hoboken). 2012;64(10):1527–1535. 22511570 10.1002/acr.21699

[22] Hammond A, et al. Job retention vocational rehabilitation for employed people with inflammatory arthritis (WORK-IA): a feasibility randomized controlled trial. BMC Musculoskelet Disord. 2017;18(1):315. 28732491 10.1186/s12891-017-1671-5 PMC5521067

[23] Madsen CMT, et al. Developing a complex vocational rehabilitation intervention for patients with inflammatory arthritis: the WORK-ON study. BMC Health Serv Res. 2023;23(1):739. 37422649 10.1186/s12913-023-09780-2 PMC10329797

[24] Boot CR, et al. One-year predictors of presenteeism in workers with rheumatoid arthritis: disease-related factors and characteristics of general health and work. J Rheumatol. 2018;45(6):766–770. 29496893 10.3899/jrheum.170586

[25] Castillo-Ortiz J, et al. Work outcome in patients with ankylosing spondylitis: results from a 12-year followup of an international study. Arthritis Care Res (Hoboken). 2016;68(4):544–552. 26414460 10.1002/acr.22730

[26] Chopp-Hurley JN, et al. Randomized controlled trial investigating the role of exercise in the workplace to improve work ability, performance, and patient-reported symptoms among older workers with osteoarthritis. J Occup Environ Med. 2017;59(6):550–556. 28379878 10.1097/JOM.0000000000001020

[27] Eichler S, et al. The effectiveness of telerehabilitation as a supplement to rehabilitation in patients after total knee or hip replacement: randomized controlled trial. JMIR Rehabil Assist Technol. 2019;6(2):e14236. 31697239 10.2196/14236 PMC6873150

[28] Sennehed CP, et al. Early workplace dialogue in physiotherapy practice improved work ability at 1-year follow-up—workup, a randomised controlled trial in primary care. Pain. 2018;159(8):1456–1464. 29554017 10.1097/j.pain.0000000000001216 PMC6085128

[29] Wynne-Jones G, et al. Effectiveness and costs of a vocational advice service to improve work outcomes in patients with musculoskeletal pain in primary care: a cluster randomised trial (SWAP trial ISRCTN 52269669). Pain. 2018;159(1):128–138. 28976423 10.1097/j.pain.0000000000001075

[30] Sloot R, et al. Reumatische aandoeningen in Nederland: Ervaringen en kengetallen (‘Rheumatic diseases in the Netherlands: experiences and key figures’). Utrecht: Nivel; 2016.

[31] Skivington K, et al. A new framework for developing and evaluating complex interventions: update of Medical Research Council guidance. BMJ. 2021;374:n2061. 34593508 10.1136/bmj.n2061 PMC8482308

[32] Tong A, Sainsbury P, Craig J. Consolidated criteria for reporting qualitative research (COREQ): a 32-item checklist for interviews and focus groups. Int J Qual Health Care. 2007;19(6):349–357. 17872937 10.1093/intqhc/mzm042

[33] Ministry of Social Affairs and Employment. Arbowet (‘Working Conditions Act’). Ministry of Social Affairs and Employment; 2017.

[34] de Jong P, Everhardt T, Schrijvershof C. Toepassing van de wet Verbetering Poortwachter (‘Application of The Dutch Eligibility for Permanent Incapacity Benefit (Restrictions) Act’). APE; 2011.

[35] de Buck PD, et al. Communication between Dutch rheumatologists and occupational physicians in the occupational rehabilitation of patients with rheumatic diseases. Ann Rheum Dis. 2002;61(1):62–65. 11779762 10.1136/ard.61.1.62 PMC1753888

[36] Conen W, Debets M. Precariousness and social risks among solo self-employed in Germany and the Netherlands. Edward Elgar; 2019.

[37] Hurkmans EJ, et al. KNGF guideline rheumatoid arthritis. 2018. https://www.kngf.nl/binaries/content/assets/kennisplatform/onbeveiligd/guidelines/reumatoide-artritis-2020/kngf-rheumatoid-arthritis-ra-2018-practice-guideline.pdf.

[38] van Weely SFE, et al. Aanbevelingen fysiotherapie bij mensen met axiale spondyloartritis (‘Physiotherapy recommendations in people with axial spondyloarthritis’). 2019. https://reumanetnl.nl/wp‐content/uploads/2019/06/Aanbevelingen‐Fysiotherapie‐bij‐mensen‐met‐AxialeSpondyloartritus.pdf.

[39] Boonen A, Lems WF. Arbeid als behandeldoel: Nieuwe richtlijn ‘Reumatoïde artritis en participatie in arbeid’ (‘Work as a treatment goal: new guideline ‘Rheumatoid arthritis and work participation’’). Nederlands Tijdschrift voor Geneeskunde, 159; 2015. https://www.ntvg.nl/system/files/publications/a9593.pdf. 26732216

[40] Fit for Work. Fit for work? Spier- en gewrichtsaandoeningen en de Nederlandse arbeidsmarkt (‘Fit for work? Muscle and joint disorders and the Dutch labour market’). 2013. https://fitforworknederland.nl/wp-content/uploads/2015/12/6_FitforWork_Quickscan_2013.pdf.

[41] Krueger RA. Focus groups: a practical guide for applied research. Sage Publications; 2014.

[42] Hutting N, et al. The effects of integrating work-related factors and improving cooperation in musculoskeletal physical therapy practice: protocol for the ‘WORK TO BE DONE’ cluster randomised controlled trial. BMC Musculoskelet Disord. 2020;21(1):360. 32513153 10.1186/s12891-020-03375-2 PMC7281957

[43] Braun V, Clarke V. Using thematic analysis in psychology. Qual Res Psychol. 2006;3(2):77–101.

[44] Morse JM, et al. Verification strategies for establishing reliability and validity in qualitative research. Int J Qual Methods. 2002;1(2):13–22.

[45] Yanow D, Schwartz-Shea P. Interpretation and method: empirical research methods and the interpretive turn. Routledge; 2015.

[46] Lewinski AA, et al. Applied rapid qualitative analysis to develop a contextually appropriate intervention and increase the likelihood of uptake. Med Care. 2021;59(6 Suppl 3):S242. 33976073 10.1097/MLR.0000000000001553 PMC8132894

[47] Nevedal AL, et al. Rapid versus traditional qualitative analysis using the Consolidated Framework for Implementation Research (CFIR). Implemen Sci. 2021;16(1):67. 10.1186/s13012-021-01111-5 PMC8252308 34215286

[48] Ward MM, et al. 2019 Update of the American College of Rheumatology/Spondylitis Association of America/Spondyloarthritis Research and Treatment Network recommendations for the treatment of ankylosing spondylitis and nonradiographic axial spondyloarthritis. Arthritis Care Res (Hoboken). 2019;71(10):1285–1299. 31436026 10.1002/acr.24025 PMC6764857

[49] Beurskens AJ, et al. A patient-specific approach for measuring functional status in low back pain. J Manipulative Physiol Ther. 1999;22(3):144–148. 10220712 10.1016/s0161-4754(99)70127-2

[50] Stevens A, et al. Ready for goal setting? Process evaluation of a patient-specific goal-setting method in physiotherapy. BMC Health Serv Res. 2017;17(1):618. 28859652 10.1186/s12913-017-2557-9 PMC5579955

[51] Lambeek LC, et al. Randomised controlled trial of integrated care to reduce disability from chronic low back pain in working and private life. BMJ. 2010;340:c1035. 20234040 10.1136/bmj.c1035 PMC2840223

[52] Lindgren L, et al. Newly diagnosed with inflammatory arthritis (NISMA)—development of a complex self-management intervention. BMC Health Serv Res. 2023;23(1):1–23. 36750937 10.1186/s12913-022-09007-w PMC9902823

[53] Salmon VE, et al. Developing a group intervention to manage fatigue in rheumatoid arthritis through modifying physical activity. BMC Musculoskelet Disord. 2019;20(1):1–94. 31054567 10.1186/s12891-019-2558-4 PMC6500086

