# Supplementary material for: Development of a Multimodal, Physiotherapist-Led, Vocational Intervention for People with Inflammatory Arthritis and Reduced Work Ability: A Mixed-Methods Design Study
Source: J Occup Rehabil. 2024 Feb 5;34(4):832–46. doi: 10.1007/s10926-023-10170-y (PMC11550277; doi:10.1007/s10926-023-10170-y)
Supplement: Supplementary file 1 — Supplementary file1 (DOCX 18 kb) [file 10926_2023_10170_MOESM1_ESM.docx]

Appendix 1

Table 3: Topics discussed in the group meetings

| **Topics** | **Group meeting 1: patient representatives (n=6)** | **Group meeting 2: patient representatives (n=10)** | **Group meeting 3: PTs (n=12)** | **Group meeting 4: (occupational) HCPs (n=9)** | **Group meeting 5: researchers (n=6)** | **Group meeting 6: PTs of the feasibility test (n=4)** |
| --- | --- | --- | --- | --- | --- | --- |
| **General topics** | | | | | | |
| Experiences with work-related problems | X | X |  |  |  |  |
| Actions performed to reduce work-related problems | X | X |  |  |  |  |
| Experiences with (not) receiving work-related support | X | X |  |  |  |  |
| Provision of work-related care (how often, why (not)?) |  |  | X |  |  |  |
| **Topics concerning the draft intervention** | | | | | | |
| Key strengths of the proposed intervention | X | X | X | X | X | X |
| Weaknesses of the proposed intervention | X | X | X | X | X | X |
| Usefulness of workplace examination |  |  | X | X | X |  |
| Referral to other (occupational) HCPs | X | X | X | X | X | X |
| Necessary adjustments/additions to the draft protocol | X | X | X | X | X | X |
| Experiences of the feasibility test |  |  |  |  |  | X |
| **Topics concerning the training of PTs in the trial** | | | | | | |
| Required competencies and skills of PTs to deliver the intervention | X | X | X | X | X | X |
| Necessary adjustments/additions to the e-learning courses |  |  | X | X | X | X |
